# Stereoscopic spatial graphical method of Mueller matrix: Global-Polarization Stokes Ellipsoid

**DOI:** 10.1007/s12200-024-00132-4

**Published:** 2024-08-16

**Authors:** Xinxian Zhang, Jiawei Song, Jiahao Fan, Nan Zeng, Honghui He, Valery V. Tuchin, Hui Ma

**Affiliations:** 1 Tsinghua Shenzhen International Graduate School, Tsinghua University, Shenzhen, 518055 China; 2 School of Teacher Education, Nanjing Normal University, Nanjing, 210097 China; 3 Institute of Physics, Saratov State University, Saratov, 410012 Russia; 4 Department of Physics, Tsinghua University, Beijing, 100084 China; 5 Tsinghua-Berkeley Shenzhen Institute, Tsinghua University, Shenzhen, 518055 China

**Keywords:** Full polarization, Mueller matrix, Tissue characterization, Optical measurement

## Abstract

A Mueller matrix covers all the polarization information of the measured sample, however the combination of its 16 elements is sometimes not intuitive enough to describe and identify the key characteristics of polarization changes. Within the Poincaré sphere system, this study achieves a spatial representation of the Mueller matrix: the Global-Polarization Stokes Ellipsoid (GPSE). With the help of Monte Carlo simulations combined with anisotropic tissue models, three basic characteristic parameters of GPSE are proposed and explained, where the *V* parameter represents polarization maintenance ability, and the *E* and *D*_†_ parameters represent the degree of anisotropy. Furthermore, based on GPSE system, a dynamic analysis of skeletal muscle dehydration process demonstrates the monitoring effect of GPSE from an application perspective, while confirming its robustness and accuracy.

**Supplementary Information:**

The online version contains supplementary material available at 10.1007/s12200-024-00132-4.

## Introduction

Compared to traditional optical measurement methods, polarimetry cannot only expands data dimensions but also improve imaging contrast by selecting specific photons [1–6]. Polarimetric parameters based on Mueller matrices and Stokes vectors can help to clarify some physical phenomena such as depolarization and anisotropy, and also can be associated with the morphology, composition, and microstructure of biological tissues, which are closely related to the pathological characteristics of biological tissues [7–11]. The evaluation indicators by polarization measurements can provide guidance and judgment basis for the characteristics analysis of biological samples and the diagnosis and treatment of diseases, such as breast cancer [12], cervical cancer [13–15], colon cancer [16, 17], skin cancer [18, 19], gastrointestinal cancer [20, 21], thyroid cancer [14, 22, 23], lung cancer [24], liver cirrhosis and cancer [25, 26], breast ductal cancer [27, 28], and large tissue [29–34].The Poincaré sphere provides an intuitive description of all Stokes vector space [17, 35–37]. Mueller matrix is a complete expression of the polarization characteristics of various materials, biological tissues, and media. Compared with Mueller matrix, Stokes vector is more singular and easily affected by the incident polarization state [38–41]. However, the visualization of the Mueller matrix is rare proposed [42]. In this study, we hope to extend the projection of Stokes vector to the projection of Mueller matrix, and then establish an improved full polarization description method to support the inherent feature analysis of measured sample from the perspective of polarization Optics more stably, comprehensively, and intuitively. This study starts with how to extend the visualization method of Stokes vectors to the Mueller matrix. By experimental observation of biological tissues including skeletal muscle, fat, and liver, the correlation between the spatial expressions of Mueller matrices and the corresponding tissue properties is revealed. Subsequently, based on Monte Carlo simulations, we independently regulate two sources of anisotropy, birefringence and cylindrical scattering, to further explore the influence and variation of tissue anisotropy on polarization space, and then extract suitable parameters to characterize the types and degrees of microscopic and optical changes in tissues. Finally, as an application demonstration, we employ this spatial descriptive form of Mueller matrix and corresponding parameters to the continuous monitoring and quantitative analysis of dynamic dehydration process of skeletal muscle. Overall, this study provides a method to intuitively describe the full polarization properties of biological tissues, especially anisotropy, in 3D space, whose capability and advantage has been illustrated and confirmed in preliminary biomedical study.

## Materials and methods

### Experimental setup and tissue samples

**Fig. 1:**
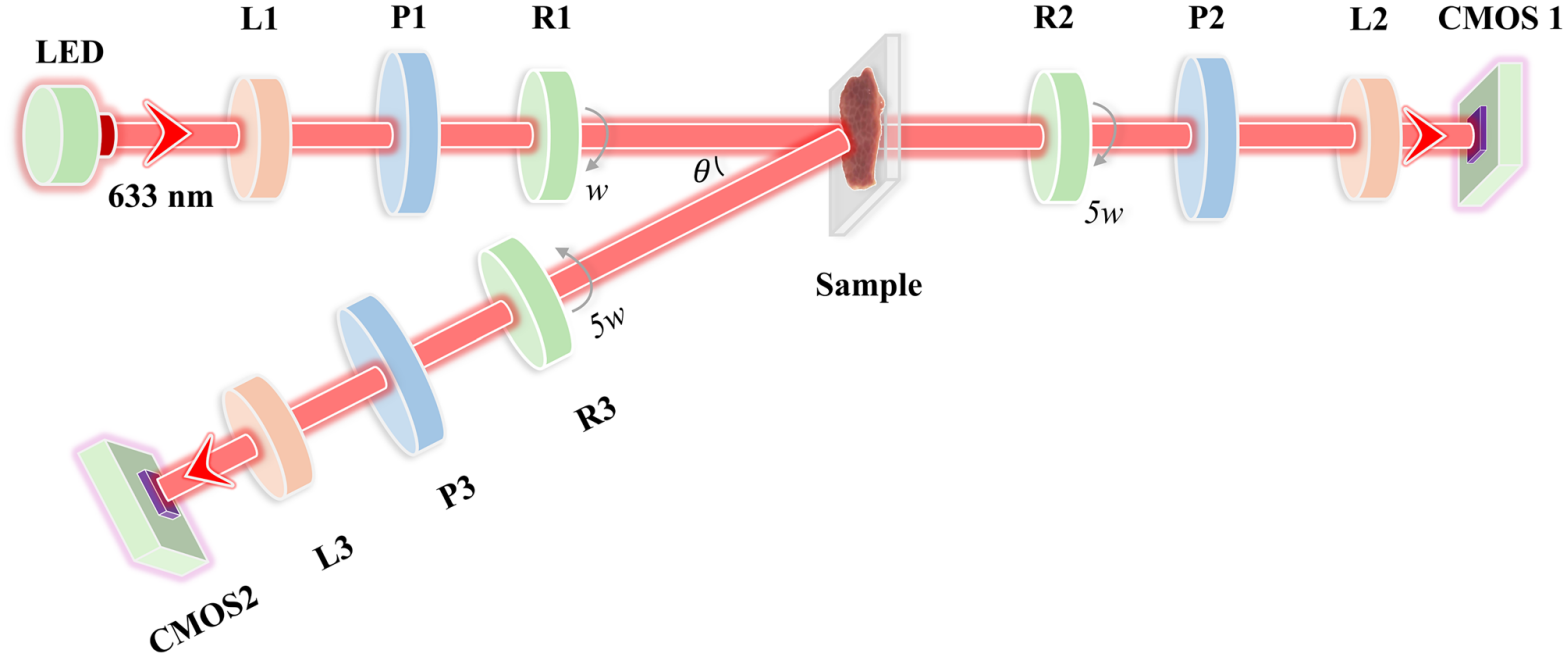
Schematic of Mueller matrix measurement setup for backscattering and transmission. The angle between PSG and PSA in the backscattering path is 15°

As shown in Fig. 1, the experimental setup is a Mueller matrix imaging system based on dual rotating retarder method [43, 44]. Considering the requirements of measurements, this setup comprises two detection pathways: forward transmission and backward scattering.

The illumination light from the LED light source (3 W, 633 nm, $\Delta \lambda$ = 20 nm, Xlamp XP-E, Cree) is initially processed through a polarization state generator (PSG). This PSG includes a lens (L1, LBTEK Optic, China) for collimating the light, a polarizer (P1, extinction ratio > 5000:1, LBTEK Optic, China), and a quarter-wave plate (R1, LBTEK Optic, China) for generating various polarization states through rotation. In the forward transmission pathway, the light is first scattered by the sample and then passed through the polarization state analyzer (PSA). The PSA consists of a rotating quarter-wave plate (R2), a polarizer (P2), and a lens (L2). For the backward scattering pathway, the light, after scattering through the sample, similarly passes through a PSA with the same structure and component models as the PSA in the forward transmission pathway. To mitigate reflections from the sample surface, the angle between the PSG and PSA in the backward pathway is set at 15°. It is noteworthy that polarizers P1, P2, and P3 are fixed horizontally, while quarter-wave plates R1, R2, and R3 are controlled by rotation servo motors (Thorlabs, PRM1Z8, USA) and rotate at a constant angular speed (*w* for R1, 5*w* for R2 and R3). Depending on different combinations of PSG and PSA, photons, after passing through the PSA, are captured by a CMOS camera (MV-CA016-10UM, 12-bit, Hikvision, China), generating a total of 30 intensity images with diverse polarization states. Using these intensity images, the Fourier coefficients ${\alpha }_{n}$ and ${\beta }_{n}$ in Eq. (1) can be calculated, enabling the computation of the 16 elements of the Mueller matrix. The Mueller matrix measurement setup is calibrated by measuring standard samples such as air and phantom tissues, with the systematic error controlled within 1%. Further details about the calibration of the Mueller matrix measurement can be found in Ref. [45].

1 $$I = \alpha_{0} + \sum\limits_{n = 1}^{12} {[\alpha_{n} \cos (2nw) + \beta_{n} \sin (2nw)]} .$$

Porcine liver, bovine skeletal muscle, and porcine fat are chosen as measurement samples for this study. Previous research has indicated that skeletal muscle exhibits significant anisotropy due to its orderly arranged fibers, while the fat is like isotropic medium. A liver sample contains both anisotropic connective tissues and isotropic hepatic lobule tissues [29]. Once the sample is extracted from the animal, it is preserved in phosphate-buffered saline (PBS) at 4 °C until needed for experiments. Prior to experimentation, the sample can return to room temperature through a resting period. Considering the thickness limitations in transmission measurements, a microtome (Leica VT1200S) was used to slice the samples into thin sections of 0.06 cm, while samples for backward detection were cut into a thickness of 0.5 cm. Additionally, all samples were cut into square pieces measuring 1.5 cm × 1.5 cm.

### Global-Polarization Stokes Ellipsoid (GPSE)

Stokes vector describes the polarization properties of light. Compared to Mueller matrix detection, Stokes vector detection is simpler and faster. Stokes vector analysis of specific samples is closely related to the selection of incident polarization state. In contrast, the Mueller matrix is more stable and unaffected by the incident polarization state and contains full polarization information connected with inherent sample characteristics.

When the Stokes vector is projected onto the Poincaré sphere, the spatial relationships of the projection points can provide a more intuitive understanding of polarization optical phenomena and processes. Inspired by this visualization method, we extend this thought to the Mueller matrix in this study.

**Fig. 2:**
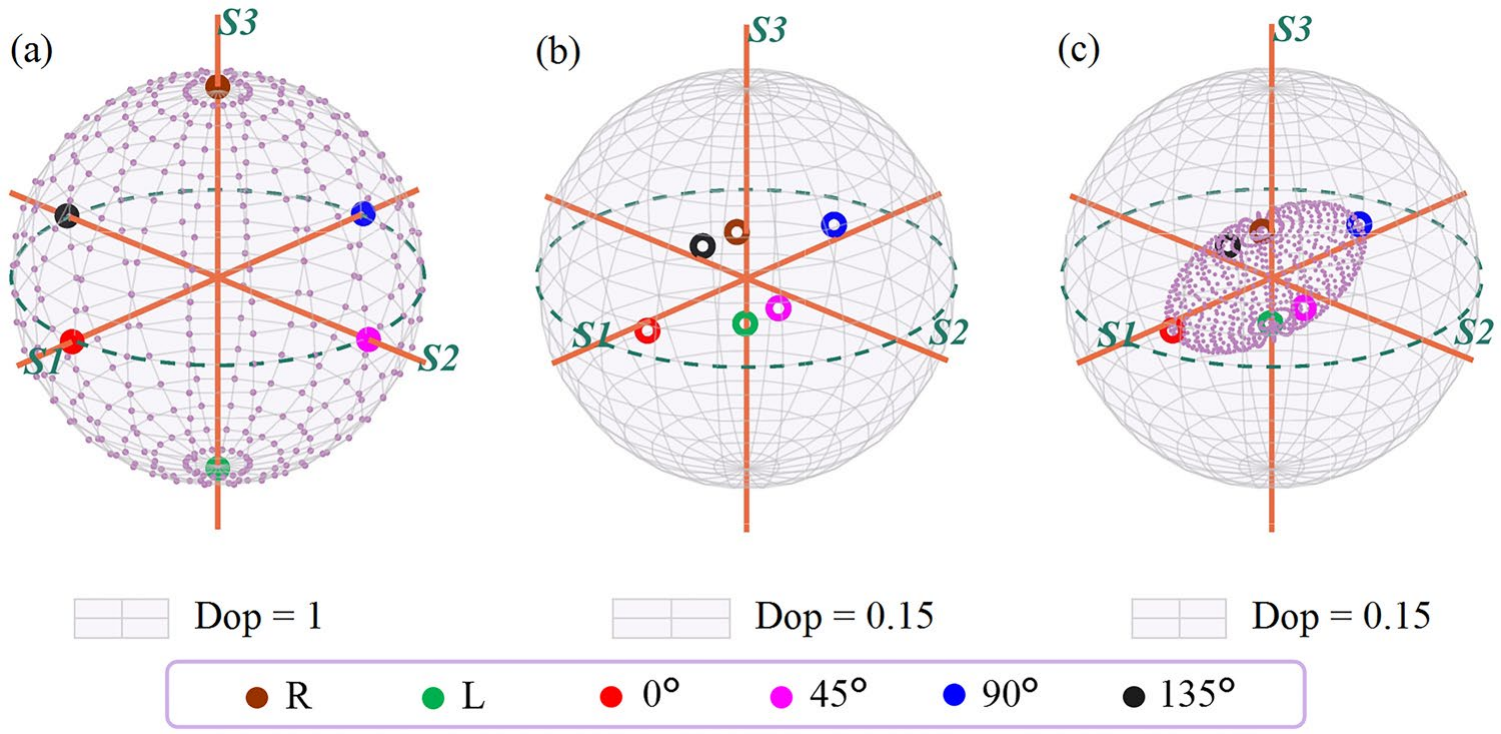
**a** Poincaré sphere system: purple dots represent fully polarized light other than the 6 mentioned states. **b** and **c** depict the projection of the 6 mentioned and all fully polarized states following interaction with bovine skeletal muscle, respectively. Additionally, the sphere in **a** corresponds to DoP of 1, while those in **b** and **c** correspond to DoP of 0.15

As depicted in Fig. 2a, the projections of linear polarizations at 0$^\circ$, 45$^\circ$, 90$^\circ$, and 135$^\circ$, as well as right and left circular polarization, on the Poincaré sphere correspond to the intersection points of the coordinate axes with the unit sphere. They are commonly chosen as incident polarization states when Stokes vector is detected.

Equation (2) delineates the interaction process between light and a medium, where $M$ symbolizes the Mueller matrix, $S$ and *S*ʹ represents the Stokes vector of the incident and exit light, respectively.

2 $$S^{\prime} = M \cdot S.$$

Here we take bovine skeletal muscle as the sample (muscle fiber orientation roughly along the *X*-axis) and measure its backward Mueller matrix using the setup in Fig. 1. After obtaining the Mueller matrix of the sample, for the six types of basic incident polarized lights, the corresponding outgoing Stokes vectors can be derived by Eq. (2). Their projections onto the Poincaré sphere are illustrated in Fig. 2b, where the external sphere’s degree of polarization (DoP) is 0.15. The projection points of the six outgoing states are evenly distributed in the interior of the Poincaré sphere, similar with the relative distribution of the incident states.

Due to the Poincaré sphere covering all complete polarization states, to establish the spatial distribution projection corresponding to a Mueller matrix, except for six basic polarization states, other types of linear polarization state on the equator and elliptic polarization states on the sphere, as depicted by the purple points in Fig. 2a, should be considered. As many points as possible on the Poincaré sphere are uniformly selected as the incident states, the projection points of the corresponding outgoing states by Eq. (2) can be shown in Fig. 2c. As long as enough polarization points on the Poincaré sphere are selected, their projection points can be connected to form a topological space representing the projection of Mueller matrix onto the Poincaré sphere system.

**Fig. 3:**
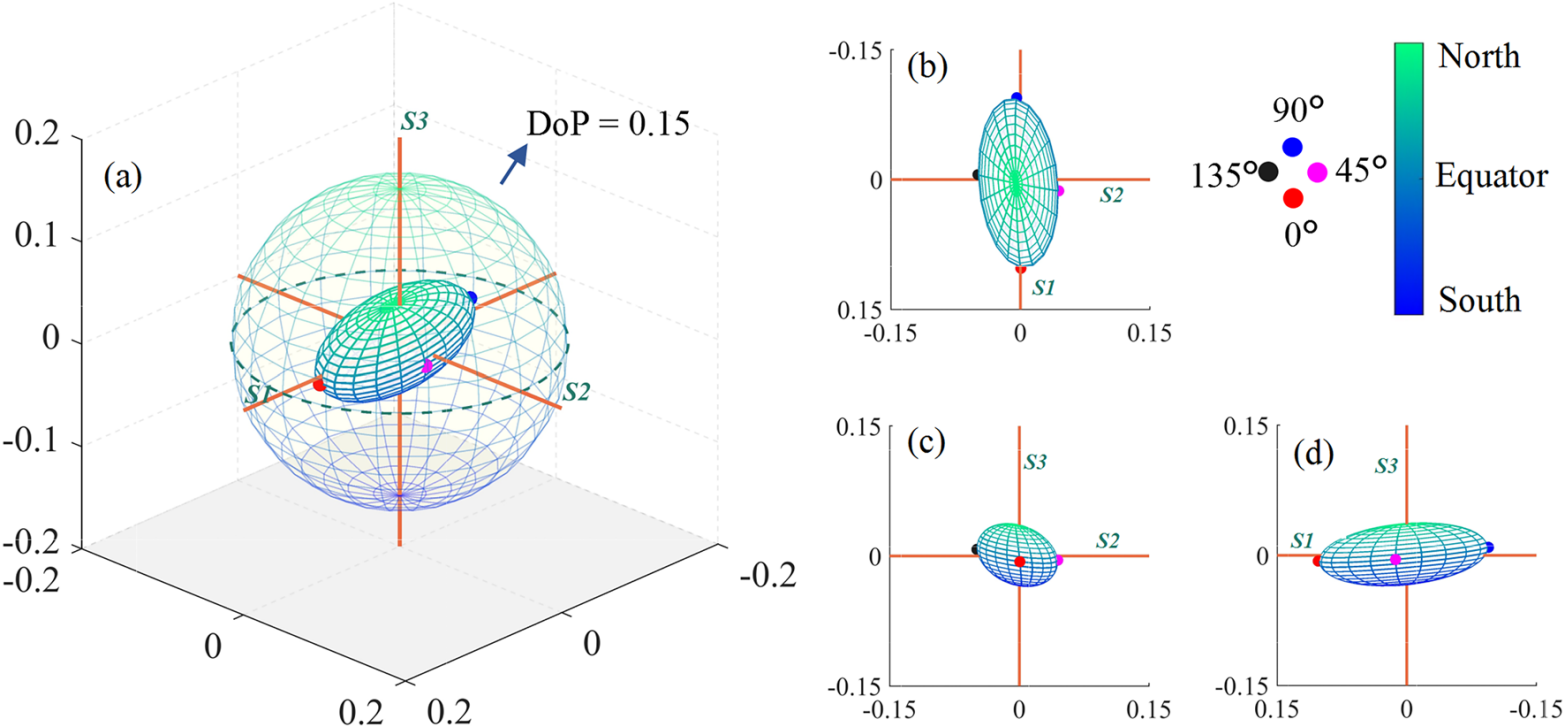
**a** GPSE of bovine skeletal muscle. **b**, **c**, and **d** show GPSE projections along the positive direction of *S3*, *S1*, and *S2* axes, respectively. The color shift from green to blue on the GPSE surface indicates light detection closer to the north pole to the south pole

For easier observation, we assigned colors to the incident states based on their corresponding latitudes on the surface of the Poincaré sphere, as illustrated in Fig. 3a. Green denotes polarization states closer to the north pole, while blue represents those closer to the south pole. Points represented in red, magenta, blue, and black correspond respectively to linearly polarized light at 0$^\circ$, 45$^\circ$, 90$^\circ$, and 135$^\circ$. Observing this figure along the positive directions of the *S3*, *S1*, and *S2* axes yields results as shown in Fig. 3b−d, respectively.

After traversing the project calculations of Stokes vectors of all complete polarization states, the projection of Mueller matrix typically exhibits an ellipsoidal shape as shown in Fig. 3. This study define this projection of the Mueller matrix as “Global-Polarization Stokes Ellipsoid (GPSE)”. Compared to directly analyzing the numerical values of Mueller matrix, this visualization GPSE system can help us intuitively understand the polarization characteristics of the medium.

### Mueller matrix polar decomposition (MMPD)

Mueller matrix elements of tissues contain all polarization information related to their optical properties and microstructure. It is difficult to achieve specific characterization of certain optical properties or structural functions based on a single matrix element. Usually, we employ parameters from matrix transformations or matrix decomposition to improve the interpretability of Mueller matrix.

The MMPD method decomposes the Mueller matrix into product of three fundamental matrices, as expressed in Eq. (3). Each of these matrices holds distinct physical meanings, where ${M}_{\text{D}}$ is a diattenuator, ${M}_{\text{R}}$ is a retarder, and ${M}_{\Delta }$ is a depolarizer.

3 $$M = M_{\Delta } \cdot M_{{\text{R}}} \cdot M_{{\text{D}}} .$$

Further parameters, such as the value of diattenuation, retardance, and depolarization can be introduced, as detailed in Eqs. (4), (5), and (6), respectively.

4 $$D = \sqrt {M_{12}^{2} + M_{13}^{2} + M_{14}^{2} } \in [0,1],$$

5 $$R = \cos^{ - 1} \left[ {\frac{{\text{tr}(M_{{\text{R}}} )}}{2} - 1} \right],$$

6 $$\Delta = 1 - \frac{{\left| {{\text{tr}}(M_{\Delta } ) - 1} \right|}}{3} \in [0,1].$$

The phase retardation in Eq. (5) comprises both linear and circular phase retardations. In this study, our discussion emphasis is the value of linear phase retardation, defined as specified in Eq. (7).

7 $$\delta = \cos^{ - 1} \left\{ {\sqrt {\left[ {M_{{\text{R}}} (2,2) + M_{{\text{R}}} (3,3)} \right]^{2} + \left[ {M_{{\text{R}}} (3,2) - M_{{\text{R}}} (2,3)} \right]^{2} } - 1} \right\}.$$

More details of the definition and derivation of MMPD parameters can be found in Ref. [7].

### Monte Carlo (MC) simulation

**Fig. 4:**
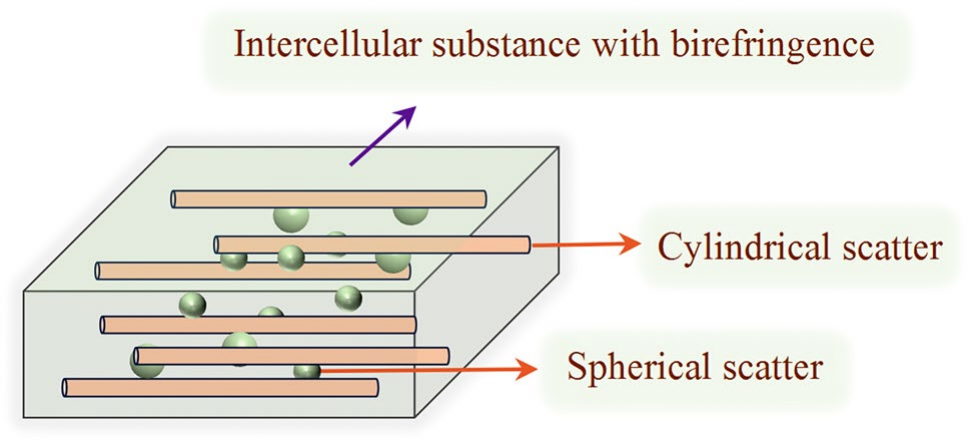
Schematic of SCBM in MC simulation

MC simulation can be used to simulate the propagation of polarized light in biological tissues. In previous studies, by the combination of polarization-sensitive MC with a sphere-cylinder birefringence model (SCBM) as illustrated in Fig. 4, we have successfully provided theoretical explanations for polarization optical phenomena in many biological tissues [46, 47].

In a SCBM model, we use spherical scatterers to simulate cell, nucleus, and other types of organelles, and use infinite cylindrical scatterers to simulate fibrous tissue like collagen and muscular fibers. We can also adjust the intrinsic anisotropy of tissue by interstitial birefringence.

Using bovine skeletal muscle as a simulated object, the parameters are set as follows [48]: the tissue thickness is 0.06 cm, the diameter of spherical scatterers and cylindrical scatterers are 0.2 and 1.5 μm, respectively. The scattering coefficients of spherical scatterers and cylindrical scatterers are 20 and 180 cm^−1^, respectively. The relative refractive indices of the spherical scatterers and cylindrical scatterers are 1.39 and 1.47, respectively. Considering the ordering of muscular fiber arrangement, the cylindrical scatterers are oriented along the *X*-axis with a full width at half maximum (FWHM) of 20$^\circ$. The refractive index difference of intercellular birefringence is 0.0003, with its optical axis along the *X*-axis. Additionally, the detection wavelength is 633 nm, and the total number of simulated photons is 10^7^.

## Results

### GPSE of biological tissue experiments

In Section 2.2, we demonstrated how to obtain the GPSE based on Mueller matrix. In this section, we will observe the correlation between GPSE and tissue characteristics.

Figure 3 shows the GPSE of bovine skeletal muscle. Each point inside the Poincaré sphere represents partially polarized light (the origin represents completely unpolarized light), and the distance from the origin corresponds to the DoP. The muscle fibers of the skeletal sample mentioned above are roughly along the *X*-axis. Theoretically, a refractive index difference appears in the axial and radial direction of the fibers. This means that if the incident polarization state is parallel or perpendicular to the fiber orientation, the light will completely enter one of the two basic channels of birefringence as ordinary ray or extraordinary ray, and a better polarization maintaining effect will appear. For other polarization states, the phase retardance by the birefringence in parallel and vertical directions to the fiber orientation will lead to significant polarization state changes.

From Fig. 3, when detecting bovine skeletal muscle, the polarization-maintaining ability (PMA) is higher for 0$^\circ$ and 90$^\circ$ linearly polarized light, while it is lower for 45$^\circ$ and 135$^\circ$ linearly polarized light and circularly polarized light. The phenomena can be explained by the theoretical analysis in the previous paragraph. According to Fig. 3b−d, it can be noticed that the incident polarization state corresponding to the maximum PMA is very close to *S1* but not completely coincident. Considering the bovine skeletal muscle is composed of muscle fibers arranged in a high degree of order, while the muscle fiber orientation may be centered on the *X* direction, and with an orientation fluctuation in the three-dimensional space. From GPSE, the PMA difference can be observed and extracted more clearly, which implies a way to analyze the orientation characteristics of fibrous biological tissues.

**Fig. 5:**
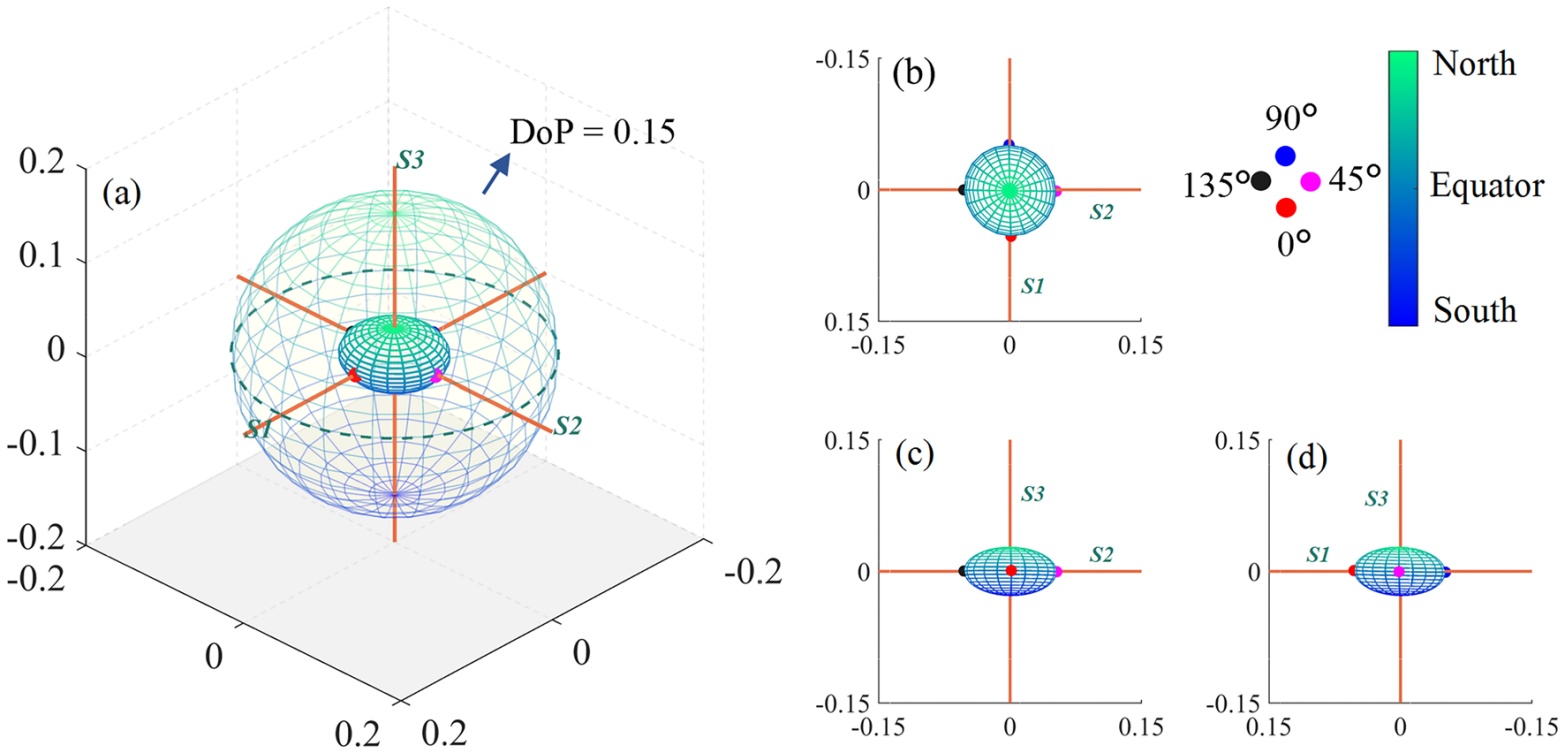
**a** GPSE of porcine fat. **b**, **c**, and **d** show GPSE projections along the positive direction of *S3*, *S1*, and *S2* axes, respectively

Unlike skeletal muscle, fat is often considered as an isotropic tissue. The GPSE of porcine fat, measured by the same setup, is shown in Fig. 5a. Although both GPSEs of fat and skeletal muscle exhibit an ellipsoidal shape, there are significant differences between them. The three symmetry axes of muscle GPSE do not completely coincide with *S1*, *S2*, and *S3*, and this deviation is likely related to the form birefringence from fibrous structures and intrinsic birefringence from intercellular spaces. If we examine the GPSE of fat, although the polarization maintenance ability of circularly polarized light in fat tissue is still weaker, unlike skeletal muscle, the PMA of various linearly polarized lights is relatively similar. The three symmetric axes of fat GPSE basically coincides with the *S1*, *S2*, and *S3*. This implies almost no linear or circular phase retardance in fat sample, meaning no typical anisotropic phenomena such as birefringence and optical rotation.

In both Figs. 3a and 5a, the outer sphere represents a DoP of 0.15. Intuitively, the volume of the GPSE for fat is smaller than that for skeletal muscle, indicating an overall weaker PMA of fat. Integrating the distance from each point on the GPSE surface to the origin provides an evaluation way of the sample’s PMA. This integration value is, in fact, the volume of the GPSE. Based on this, we define a parameter *V* to characterize the sample’s PMA, as shown in Eq. (8).

8 $$V = \sqrt[3]{{a \cdot b_{{\dag }} \cdot c}} \in [0,1].$$

In Eq. (8), $a$, *b*_†_, and $c$ represent the lengths of the long, middle, and short axis of the GPSE, respectively. When *V* is equal to 1, the GPSE is a unit sphere, meaning complete polarization maintenance. When *V* is equal to 0, the GPSE collapses to a point, indicating complete depolarization.

**Fig. 6:**
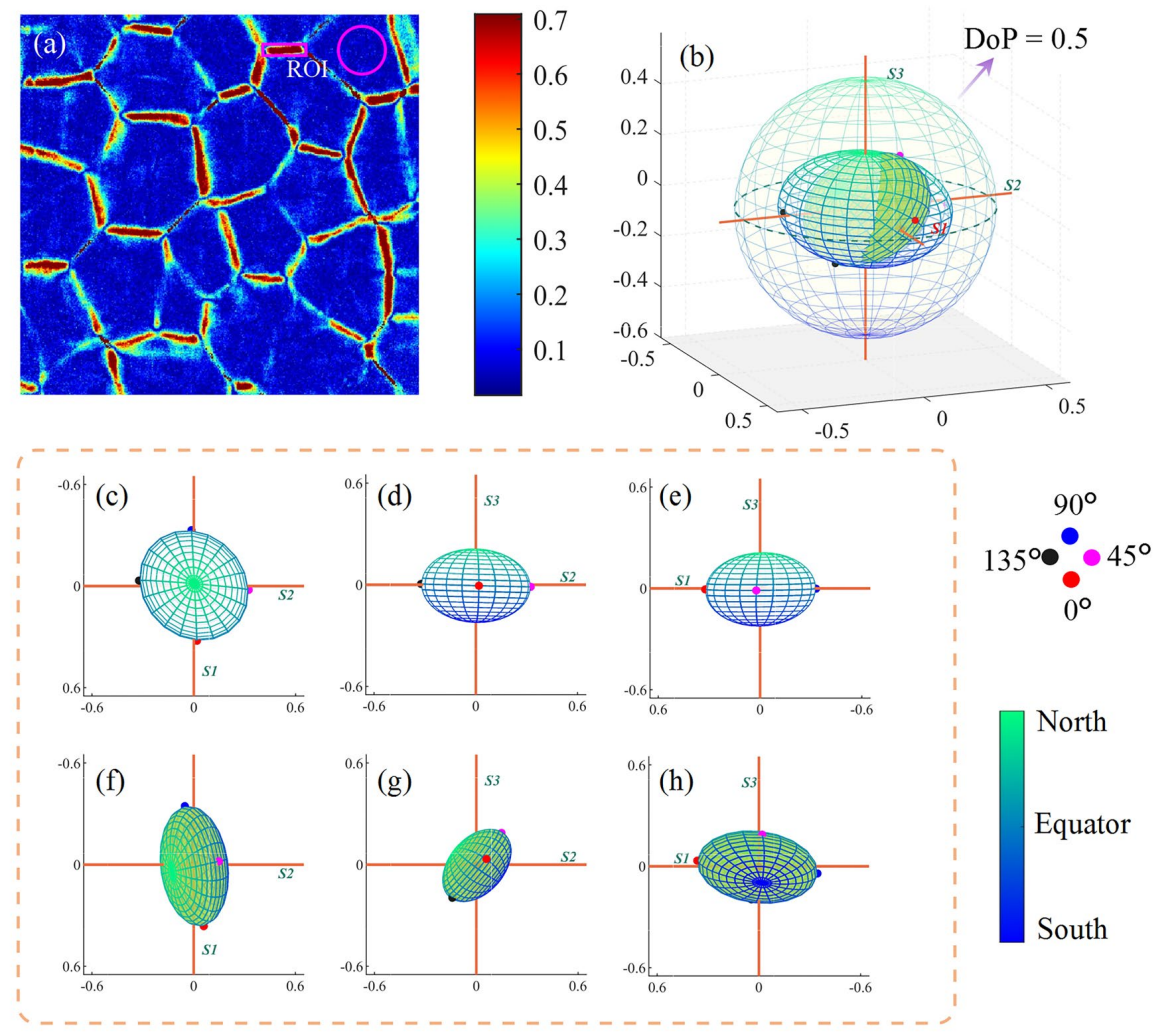
**a** $\delta$ parameter distribution of porcine liver. **b** GPSE of hepatic lobule and connective tissue, with outer GPSE corresponding to hepatic lobule. **c**−**e** and **f**−**h** show GPSE projections along the *S3*, *S1*, and *S2* axes for hepatic lobule and connective tissue, respectively

Next, polarization imaging was performed on a liver tissue. As a common biological tissue, the liver serves various functions, including glycogen storage, plasma protein synthesis, hormone production, detoxification, and other physiologic processes [29]. In contrast to both muscle and fat, liver tissue simultaneously includes isotropic hepatic lobule tissues and anisotropic connective tissues. The distribution of linear phase retardation *δ* of the liver sample is illustrated in Fig. 6a The value of $\delta$ in the hepatic lobule region is relatively small, while the value is larger in the connective tissue region. To investigate the GPSE of hepatic lobules and connective tissue separately, we segmented the local regions of two types of tissues from the linear phase retardance image of the liver sample. The region outlined by the rectangle in Fig. 6a (approximately arranged along the *X*-axis) is selected as a typical connective tissue area. The circular frame marked typical hepatic lobular area. Based on the mean Mueller matrixes of selected region, we obtain the GPSEs of hepatic lobule tissues and connective tissues, as shown in Fig. 6b. Figure 6c−e and Fig. 6f−h respectively show the 2D projections of GPSE on *S3*, *S1*, and *S2* axes for the hepatic lobule and connective tissues.

According to Fig. 6c, similar to isotropic tissues like porcine fat, the two-dimensional projection of hepatic lobular GPSE on the plane of *S1* and *S2* is approximately a circle, implying relatively consistent PMA for various linearly polarized light. In contrast, in Fig. 6f, the projection of GPSE for connective tissues forms an ellipse, similar to anisotropic tissues like bovine skeletal muscle. The long axis of GPSE from the liver sample is roughly oriented along *S1* axis, which can also be explained by the micro orientation of selected area. Furthermore, although the connective tissue shows whole anisotropy, Figs. 6g and 3c seem very different, implying the differences in the source of anisotropy between the two tissue types. For example, previous studies indicated that skeletal muscle respectively exhibits obvious form birefringence and material birefringence in the axial and radial directions, making the PMA of 45$^\circ$ or 135$^\circ$ linearly polarized state greater than that of the circular polarization state [49]. In addition, the skeletal muscle shows nonnegligible diattenuation, while the connective tissue basically does not. The research on this phenomenon remains to be further explored.

Kunnen et al. and Ivanov et al. distinguished tissue types based on the projection of Stokes detection results on Poincaré sphere [17, 35]. However, it should be noted that the selection of incident polarization state can significantly affect the contrast of Stokes images. As shown in Fig. 6b, the spatial distance in GPSE system represents the polarization contrast of different tissue samples under the same incident polarization state. For example, the shorter distance on GPSE indicates a lower discrimination on polarized images when the liver lobule and connective tissue are detected by 0$^\circ$ or 90$^\circ$ linearly polarized light. If we employ the 45$^\circ$ or 135$^\circ$ linearly polarized state, apparently the polarization image contrast between them will be enhanced effectively. Through GPSE, we can intuitively compare the amount of polarization change in different tissues and select the optimum incident polarization state to distinguish tissue characteristics.

### GPSE characteristics based on MC simulations

In Section 3.1, we observed the experimental GPSEs of several biological tissues, including skeletal muscle, fat, and liver. The skeletal muscle and the connective tissues of the liver are considered as anisotropic tissues, while the fat and the hepatic lobule tissues are approximately isotropic. In this section, we will further explore the relationship between GPSE and tissue characteristics combined with MC simulations of polarized light backscattered in tissues.

The interaction process between tissues and light can be simulated using MC program. Using SCBM, we can independently regulate the two main sources of tissue anisotropy, that is, intrinsic birefringence and form birefringence due to cylindrical scattering.

**Fig. 7:**
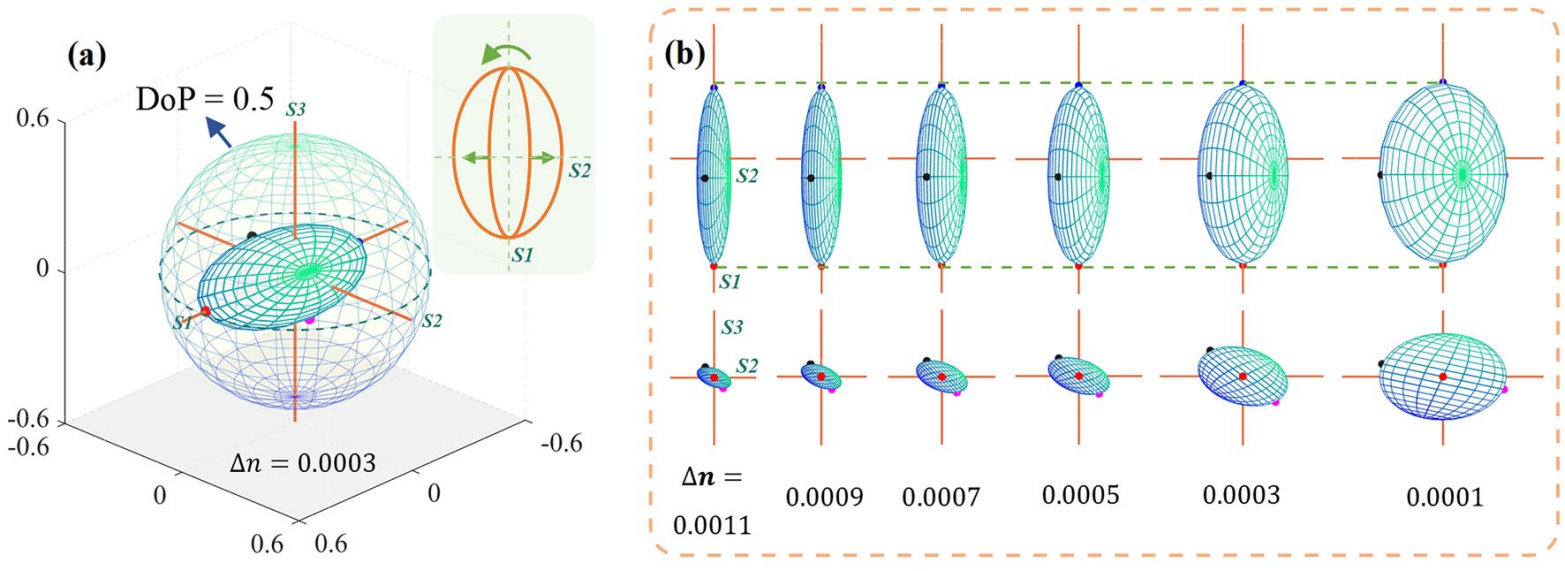
**a** GPSE of basic parameters for bovine skeletal muscle in MC simulation. **b** shows how GPSE responds as the refractive index difference decreases from 0.0011 to 0.0001. The upper and lower halves represent projections along the *S3* and *S1* axes, respectively. Further, the top right corner of **a** illustrates the evolution of GPSE along the *S3* axis

We still take bovine skeletal muscle as the reference sample for MC simulation, and the tissue thickness is set as 0.06 cm to ensure the suitable DoP. The FWHM of the cylindrical scatterers is used to describe the fiber disorder, and the percentage of cylindrical scattering coefficient to the total scattering coefficient (the total scattering coefficient is 200 cm^−1^) represents the fiber content. The GPSE shown in Fig. 7a corresponds to the basic parameters of skeletal muscle ($\Delta n$ = 0.0003, fiber content = 80%, fiber FWHM = 10°), using the same legend as above.

First, we regulate the intrinsic birefringence from 0.0011 to 0.0001 to mimic the decreasing tissue anisotropy. The two groups of 2D projections of GPSE are shown in Fig. 7b. The scales for all coordinate systems are the same, and from left to right, they correspond to refractive index differences of 0.0011, 0.0009, 0.0007, 0.0005, 0.0003, and 0.0001. It can be seen that the volume of GPSE increases with decreasing birefringence, meaning additional depolarization induced by birefringence. Compared with the long axis, the other two axes of GPSE extend significantly, making the GPSE more closely to a sphere. For the skeletal muscle, the closely aligned muscle fibers are the major anisotropic factor. Therefore, when the fiber orientation is unchanged, the PMA of linear polarized parallel to the muscle fibers, that is, along *S1* direction, is basically not affected by birefringence. The decrease of birefringence in the simulations means the less linear phase retardance on the circular or 45° linear polarized light, which corresponds to the rotation of the *S2*−*S3* projection plane around *S1*, and also shows that the reduced birefringence improves the overall tissue polarization maintaining.

**Fig. 8:**
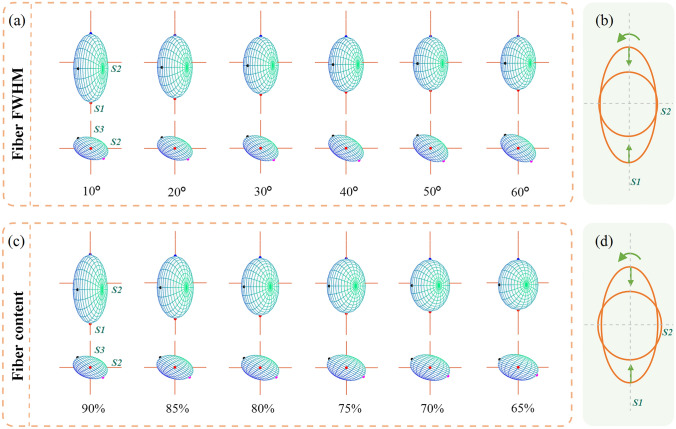
**a** and **c** depict GPSE projections as fiber FWHM increases from 10° to 60° and fiber content decreases from 90% to 65%, respectively. Upper parts represent projections along *S3* axis, lower parts along *S1* axis. **b** and **d** illustrate the evolution of GPSE projection along *S3* axis when regulating fiber FWHM and fiber content, respectively

Subsequently, we employed a different way to adjust the model anisotropy. In the second simulations, the FWHM of cylindrical scatterer distribution is increased to reduce the ordering of fiber arrangement, thereby reducing the degree of anisotropy. Here, we increased the FWHM from 10° to 60°. The projections of GPSE along the positive directions of *S3* and *S1* are shown in Fig. 8a, corresponding to the FWHM from left to right as 10$^\circ$, 20$^\circ$, 30$^\circ$, 40$^\circ$, 50$^\circ$, and 60$^\circ$. Next, the decrease of tissue anisotropy was simulated by the decrease of fiber content. Figure 8c shows the projections of GPSE along the positive direction of *S3* and *S1* axes, from left to right, corresponding to the fiber content setting of 90%, 85%, 80%, 75%, 70%, and 65%.

As illustrated in Fig. 8b and d, both the increasing fiber FWHM and the less fiber content significantly shrinks the long axis of GPSE, while the other two axes are basically unchanged. From the perspective of light scattering, both these two factors reduce the tissue anisotropy, resulting in the reduced difference between the linearly polarized light parallel or perpendicular to the cylinders and other incident polarization states. More spherical scattering probability and more disordered spatial orientation will cause more polarization fluctuations for 0$^\circ$ and 90$^\circ$ linearly polarized light, which can explain the shortened long axis of GPSE.

GPSE system is a triaxial ellipsoid, from Fig. 8b and d, the weakened cylindrical scattering causes a decreased eccentricity of GPSE. According to Fig. 7, the weakened birefringence also can decrease the eccentricity of GPSE. According to Eq. (9), the parameter *E* is defined to describe the eccentricity of GPSE, and then investigate its association with tissue anisotropy. In Eq. (9), $a$, *b*_†_, and $c$ represent the lengths of the long, middle, and short axes of the GPSE, respectively.

9 $$E = \sqrt {1 - \frac{{b_{{\dag}}^{2} + c^{2} }}{{2a^{2} }}} \in [0,1].$$

The *E* parameter reflects the PMA difference of various fully polarized states in media. When *E* is 0, GPSE appears as a spherical shape, meaning the consistent PMA of all fully polarized states. *E* closer to 1 corresponds to obvious PMA difference, which may be due to the anisotropy of light transmission or scattering processes in the medium.

It should be noted that for the case of decreased cylindrical scattering, the center of GPSE moves closer to the origin of coordinate system. We define a *D*_†_ parameter by Eq. (10) to describe the movement of GPSE ellipsoid center, which may be also related to the tissue anisotropy. In Eq. (10), $a$, *b*_†_, and $c$ represent the length of the long, middle, and short axes of GPSE, respectively, while $\left({x}_{0},{y}_{0},{z}_{0}\right)$ denotes the coordinates of GPSE center in the Poincaré sphere system. Dividing by the average of three ellipsoidal axes here is to suppress the influence of sample depolarization characteristics.

10 $$D_{{\dag}} = \frac{{3\sqrt {x_{0}^{2} + y_{0}^{2} + z_{0}^{2} } }}{{a + b_{{\dag }} + c}}.$$

**Fig. 9:**
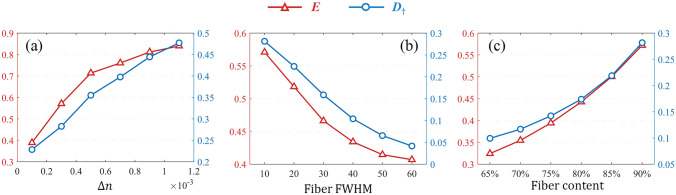
Regulation of anisotropy using GPSE parameters. The left vertical axis corresponds to the *E* parameter, while the right corresponds to the *D*_†_ parameter. **a** Regulating the refractive index difference, corresponding to Fig. 7b. **b** Regulating fiber FWHM, corresponding to Fig. 8a. **c** Regulating fiber content, corresponding to Fig. 8c

Figure 9 illustrates the MC simulation results of *E* and *D*_†_ parameters under the above three anisotropic regulation mechanisms. Figure 9a−c show the simulations with the increasing birefringence, fiber disorder, and fiber content, respectively. In each subplot, the left and right vertical axes correspond to the values of *E* and *D*_†_, respectively.

According to Fig. 9, whether the enhancement of birefringence effect, the increase of fiber arrangement order, or the increase of fiber content, will lead to the increase of *E* and *D*_†_ in MC simulations. It is clear that *E* and *D*_†_ parameters were positively correlated with the degree of anisotropy.

Table 1 lists the experimental values of GPSE parameters and MMPD parameters for skeletal muscle, fat, hepatic lobules (liver), and connective tissues (liver). As mentioned earlier, the *V* parameter of GPSE is closely related to the PMA of the sample, while the $\Delta$ parameter of MMPD represents the depolarization. Therefore, both *V* and Δ can indicate the PMA ranking of these tissues is hepatic lobules > connective tissues > skeletal muscle > fat. In the GPSE parameter system, both the *E* and *D*_†_ parameters of skeletal muscle are significant, followed by the connective tissues. By examining the MMPD parameter system, the $\delta$ parameter of connective tissues is more significant, while the *D* parameter of skeletal muscle has a maximum value. In previous studies, skeletal muscle has both form birefringence from muscle fibers and intercellular material birefringence, and also show obvious diattenuation, corresponding to greater differences in the scattering and transmission process of different incident polarization states. From this point of view, the *E* and *D*_†_ parameters of GPSE clearly indicates the global anisotropy of skeletal muscle. On the other hand, the amount of phase retardance obtained by MMPD is limited by 2π periodicity, meaning its value cannot be guaranteed to be completely positively correlated with the degree of tissue anisotropy. Therefore, in contrast, the relationship between the GPSE parameters and tissue anisotropy may be more direct and stable. Besides, the E parameters of hepatic lobules and fat are approximately equal, while the hepatic lobules may have a higher degree of anisotropy due to its larger *D*_†_.

**Table 1:** Values of GPSE and MMPD parameters for bovine muscle, porcine fat, and porcine liver

Tissues	GPSE			MMPD		
	*V*	*E*	*D*_†_	$\Delta$	$\delta\; \left(\text{rad}\right)$	*D*
Muscle	0.047	0.663	0.379	0.939	0.748	0.022
Fat	0.042	0.226	0.021	0.956	0.007	0.001
Hepatic lobules (liver)	0.303	0.225	0.030	0.692	0.026	0.011
Connective tissues (liver)	0.238	0.433	0.049	0.749	0.983	0.010

### Dehydration process monitoring based on GPSE system

Whether a Mueller matrix is obtained from forward or backward measurements, GPSE can serve as a graphical representation. In this section, we employ a forward imaging setup to continuously monitor the dynamic dehydration process of bovine skeletal muscle. Considering the impact of sample thickness on light intensity, the samples were uniformly sliced to a thickness of 600 μm to ensure sufficient light penetration. To expedite the tissue dehydration process, a fan was employed to enhance air flow. Utilizing the forward experimental schematic in Fig. 1, we conducted six repeated experiments, each of which a bovine skeletal muscle sample is monitored (with muscle fibers oriented along the *X*-axis) every 5 min over a period of 90 min.

**Fig. 10:**
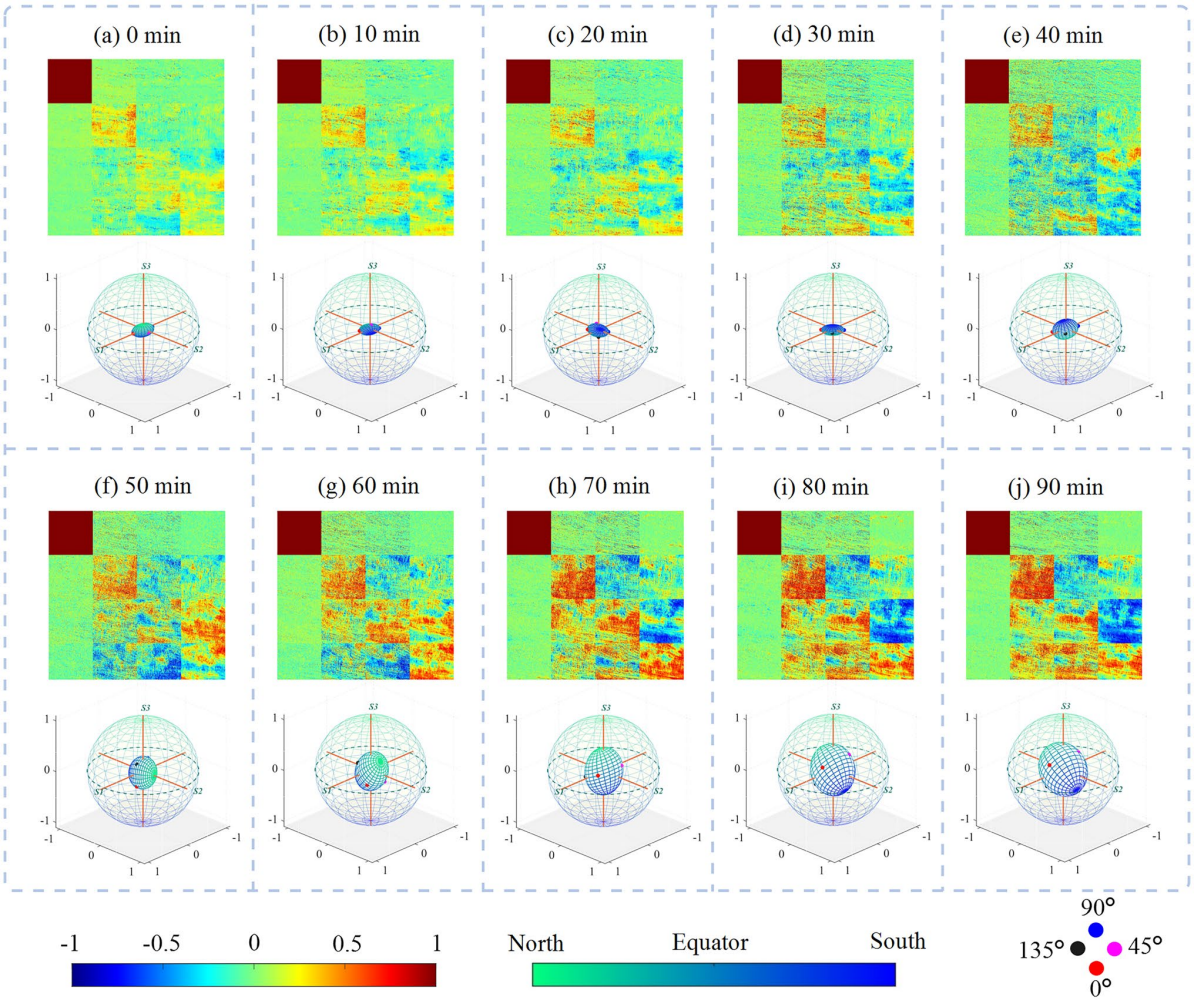
Mueller matrix and GPSE of the dehydration process for bovine skeletal muscle. **a**−**j** represent the initial and final states, with a 10-min interval

The dehydration process of bovine skeletal muscle is presented in Fig. 10, where the upper portion of each subplot is the Mueller matrix images (normalized by the m11 element), and the lower portion exhibits the corresponding GPSE. The time interval for Fig. 10a−j is set at 10 min. For intuitively track the polarization status during the dehydration process, the animation (Online Resource 1, see the Supplementary Information) provides a dynamic demonstration of the measured image sequence, corresponding to changes in the Mueller matrix and GPSE every 5 min.

From Fig. 10, it is evident that the diagonal elements of Mueller matrix are gradually enhanced during dehydration, meaning an increased tissue’s PMA. Compared with the matrix elements, the increased volume of GPSE can more intuitively indicate the changes of PMA. According to our previous study on the stages of tissue dehydration [50], at the microscopic level, dehydration may lead to tighter fiber arrangement and increased birefringence. The former makes the linear polarized light parallel or vertical to the muscle fibers easier to maintain the degree of polarization, while the latter will cause more depolarization effect in the tissue. The GPSE changes in Fig. 10 suggests that the depolarization effect is least significant in the middle stage of dehydration, corresponding to a rapid increase of *V* parameter, which may imply that the regulation of fiber arrangement by dehydration is mainly at this stage.

**Fig. 11:**
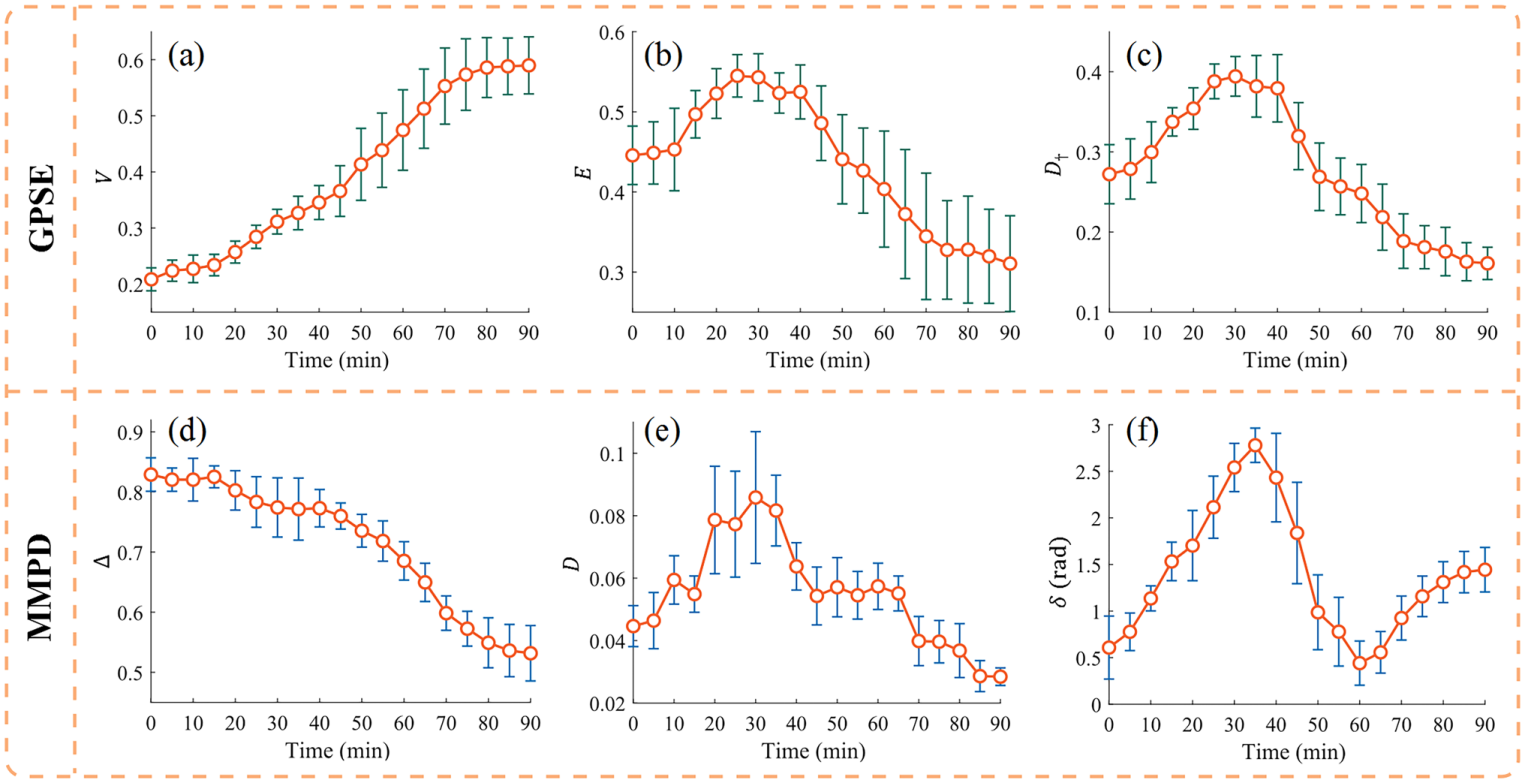
Characterization of the dehydration process for bovine skeletal muscle using the GPSE and MMPD parameter systems. **a**−**c** Correspond to the *V*, *E*, and *D*_†_ parameters of GPSE, respectively. **d**−**f** Correspond to the $\Delta$, *D*, and $\delta$ parameters of MMPD, respectively

Figure 11 shows the parameter changes from GPSE and MMPD during the dehydration experiment of bovine skeletal muscle, where (a), (b), and (c) correspond to the *V*, *E*, and *D*_†_ parameters of GPSE, while (d), (e), and (f) correspond to the depolarization, diattenuation, and linear phase retardance parameters of MMPD, respectively. Consistent with the above analysis, both the *V* parameter of GPSE and the ∆ parameter of MMPD indicate the gradual PMA increase of bovine skeletal muscle during dehydration and tends to stabilize in the later stage.

According to Fig. 11, all three parameters of *E*, *D*_†_, and *D* initially showed an increasing trend, and then reached their maximum values after about 30 min, and then gradually decrease over time. Therefore, the anisotropy of bovine skeletal muscle shows an overall trend of first increasing and then decreasing during dehydration. The microscopic tissue features in the early stage of dehydration, including the more compact arrangement of fibers and increasing birefringence, have been mentioned in our previous research. Combined with the simulation work in the previous section, the enhancement of tissue anisotropy can be explained. In the later stage of dehydration, with the significant decrease in tissue thickness and the phenomenon of light transparency, the tissue scattering coefficient decreases significantly. This may mean that the refractive index difference decreases with the tissue, leading to a decrease in the degree of anisotropy.

**Fig. 12:**
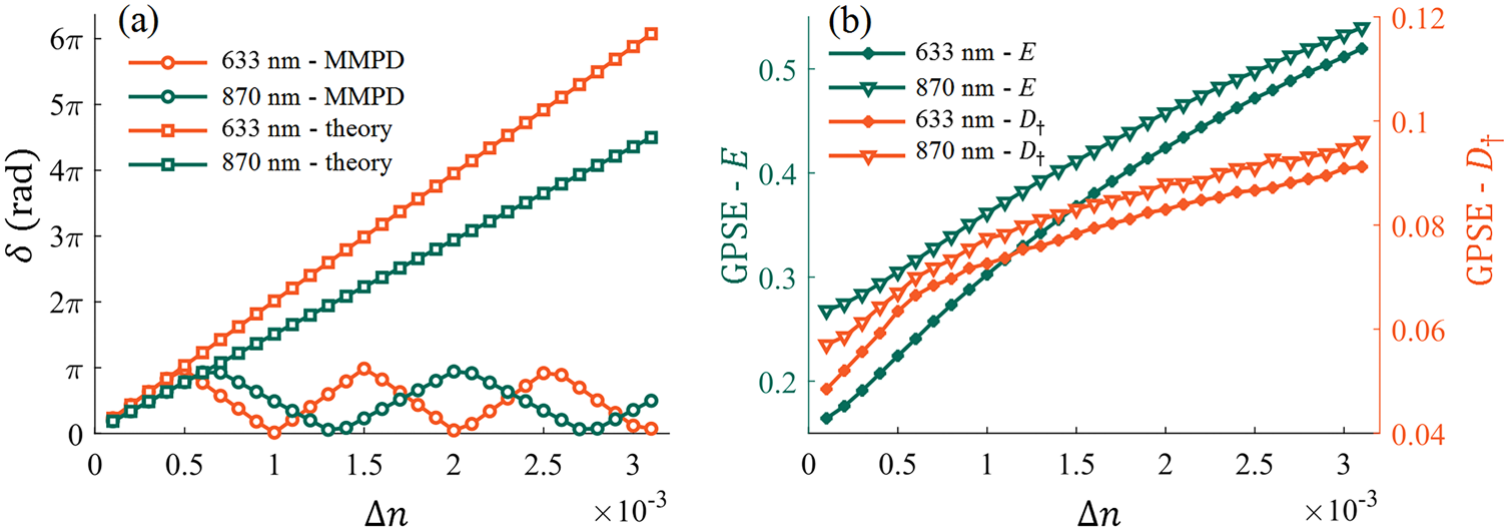
Refractive index difference increases from 0.0001 to 0.0031 at two detection wavelengths, 633 and 870 nm. **a** MMPD $\delta$ parameter: circles for direct calculations, rectangles for theoretical values after dual-wavelength unwrapping. **b** *E* parameter and *D*_†_ parameter of GPSE, corresponding to the left and right vertical axes, respectively

The major difference between GPSE and MMPD system can be observed in Fig. 11c and f, where the $\delta$ parameter manifested as an anomalous oscillation phenomenon. Considering the strong birefringence characteristics of skeletal muscles, and our previous work [51], we explain this abnormal phase retardance using the phase wrapping effect in the following Fig. 12.

During dehydration, the right and bottom edges of Mueller matrix exhibit periodic changes, which may be due to the periodic variation of phase retardance extracted by MMPD with the increasing birefringence, that is, the phase wrapping phenomenon caused by a true phase delay greater than 2π. To solve this problem, we have proposed a dual-wavelength unwrapping scheme to correct the phase retardance [51]. To illustrate this point, based on the tissue optical model of bovine skeletal muscle, we simulated the increasing birefringence from 0.0001 to 0.0031 in steps of 0.0001 at two detection wavelengths, 633 and 870 nm. Figure 12a shows the linear phase retardance values obtained directly using MMPD and corrected by the unwrapping method, respectively. It can be observed that the corrected results can reasonably increase linearly with birefringence. However, this solution requires the introduction of an additional wavelength switching module. Therefore, for the description of birefringence induced phase retardance, a characterization indicator not affected by the phase wrapping effect can be more applicable than the traditional MMPD parameter.

If we examine the dynamic GPSE during the tissue dehydration process, it rotates around the *S3* axis and accumulates a complete cycle after about 70 min. The rotation most likely originates from the linear phase retardation caused by birefringence. A full rotation cycle means the accumulated linear phase retardance of 2π. The left and right vertical axes in Fig. 12b correspond to the *E* and *D*_†_ parameter values of GPSE system, respectively. It can be seen that both *E* and *D*_†_ parameters are monotonically positively correlated with birefringence. Compared with the phase retardance parameter from MMPD, the two anisotropy parameters in GPSE system do not involve the phase wrapping effect, and can directly reflect the actual phase retardance due to tissue anisotropy without the introduction of additional dual wavelength measurements.

Taking the dehydration process of bovine skeletal muscle as an example, the GPSE characterization system demonstrated its effectiveness and robustness in tracking and extracting tissue microphysical properties. GPSE system can more stably and accurately extract anisotropic related parameters from biological tissues. GPSE can provide an important and reliable detection basis for further detailed evaluation of physiologic mechanisms in tissue processes, such as birefringence effect and fiber orientation distribution.

## Conclusions

In this study, we propose a stereoscopic spatial graphical method of Mueller Matrix by projecting the outgoing polarization states of all fully polarized light in the tissue on the Poincaré sphere, named as GPSE. Experimental findings reveal notable distinctions in the spatial and morphological characteristics of GPSEs of various tissues, such as skeletal muscle, fat, and liver. This study separately extracted characteristic parameters corresponding to GPSE volume, eccentricity, and center point position, and preliminary research demonstrated the ability of these parameters to distinguish tissue microstructures and especially anisotropy. Furthermore, through MC simulations, we respectively regulate the intrinsic birefringence, the fiber content and the orientation distribution width, revealing a robust correlation between GPSE parameters and tissue anisotropy. Finally, using the dehydration process of bovine skeletal muscle as a demonstration case, the GPSE System shows its dynamic monitoring effect. It should be pointed out that, unlike the traditional phase retardance item $\delta$ of MMPD, GPSE parameters can accurately evaluate the tissue anisotropy during dehydration without being affected by the phase wrapping effect. This study not only provides a new perspective to perceive polarization characteristics, but also enables a more intuitive identification of the optimal incident polarization state for improving the discrimination among different tissue types or during a tissue dynamic process.

### Supplementary Information

Below is the link to the electronic supplementary material.

Supplementary Material 1.

## Data Availability

Data underlying the results presented in this paper are not publicly available at this time but maybe obtained from the authors upon reasonable request.
